# Photonic and Optoelectronic Devices and Systems, Second Edition

**DOI:** 10.3390/mi16010079

**Published:** 2025-01-11

**Authors:** Muhammad A. Butt

**Affiliations:** Institute of Microelectronics and Optoelectronics, Warsaw University of Technology, Koszykowa 75, 00-662 Warsaw, Poland; ali.butt@pw.edu.pl

## 1. Introduction

Photonic and optoelectronic devices and systems are at the forefront of modern technology, enabling the precise manipulation of light for a wide range of applications [1,2,3,4]. These systems integrate key components such as lasers, LEDs, photodetectors, and optical fibers to facilitate communication, sensing, and imaging [3,5,6,7,8]. Photonic devices are pivotal in high-speed data transmission, forming the backbone of the Internet through fiber-optic networks [9]. Optoelectronic systems, which seamlessly combine electronic and optical functionalities, are vital in solar energy harvesting, medical diagnostics, and advanced manufacturing [10,11]. Together, they drive innovation across diverse fields, from telecommunications to quantum computing and autonomous technologies [12,13,14]. This Special Issue, “Photonic and Optoelectronic Devices and Systems, Second Edition”, covers a wide range of topics, featuring 11 original research papers and 1 comprehensive review, highlighting the latest advancements and trends in the field. In this editorial, a brief overview and discussion of the key insights presented in these papers is provided.

## 2. Logic Gates

Optical computing offers unique benefits, including a high bandwidth and minimal loss, making it essential for applications in signal processing, communication, and sensing [15]. Traditional optical logic gates, relying on nonlinear fibers and optical amplifiers, face challenges such as limited robustness and large sizes, restricting their integration into on-chip systems [16]. To overcome these limitations, all-optical logic gates leveraging topological photonic crystals have emerged as a promising solution for developing compact and resilient optical computing systems. Extending topological photonic crystal logic gates from single to dual operating bands enables higher throughput and significantly enhances parallel computing performance. The study “Dual-Band High-Throughput and High-Contrast All-Optical Topology Logic Gates”, conducted by Zhang et al., utilized the topological protection of valley photonic crystals combined with linear interference effects to design and implement seven optical logic gates on a silicon platform [16]. These dual-band topological photonic crystal-based gates included OR, XOR, NOT, NAND, NOR, and AND functions. The robustness of the OR gates was validated under boundary defect conditions. The findings demonstrated that topological photonic crystals could enable multi-band parallel computing in all-optical logic gates, achieving high resilience. The proposed gates have substantial potential to advance optical signal processing, communication, sensing, and related applications.

## 3. Optical Fiber Sensors and Filters

In the paper “A Novel MZI Fiber Sensor with Enhanced Curvature and Strain Sensitivity Based on Four-Core Fiber”, Zhu et al. presented a highly sensitive Mach–Zehnder interferometer (MZI) fiber sensor designed for curvature and strain measurements. The sensor design incorporates a combination of no-core fibers (NCFs) and four-core fibers (FCFs) to enhance performance [17]. The sensor was fabricated through the direct splicing of a segment of FCF between two NCF segments using an optical fiber fusion splicer. Both the FCF and NCF were composed of SiO_2_, with the FCF providing multi-path interference for enhanced sensitivity. The self-focusing property of the NCF facilitated the excitation of higher-order modes, which then split and propagated through the four cores of the FCF. The experimental results demonstrated that the sensor achieved a maximum curvature sensitivity of −78.04 dB/m^−1^ within a range of 0.0104 m^−1^ to 0.1515 m^−1^, with excellent linearity (~0.99). Strain response testing reveal4r a maximum sensitivity of −6.49 pm/με in the range of 0 to 600 με. These findings highlighted the sensor’s exceptional performance in curvature and strain detection, making it suitable for a variety of sensing applications.

In the paper “High-Sensitivity Curvature Fiber Sensor Based on Miniature Two-Path Mach-Zehnder Interferometer”, Wu et al. introduced an innovative high-sensitivity curvature fiber sensor utilizing a miniature two-path Mach–Zehnder interferometer (MTP-MZI) [18]. The sensor was established via coupling and fusing multimode fibers (MMFs) and single-mode fibers (SMFs) using arc fusion technology (AFT), producing a compact two-path MZI structure at the centimeter scale. This innovative coupling approach reduces the sensor’s size, simplifies production, and lowers manufacturing costs. In curvature sensing experiments, the MTP-MZI sensor achieved a remarkable maximum curvature sensitivity of −96.70 dB/m^−^^1^ within the range of 0.0418 m^−^^1^ to 0.0888 m^−^^1^, making it one of the most sensitive intensity-modulated curvature sensors. For temperature sensing, the sensor demonstrated a maximum sensitivity of 212 pm/°C between 30 °C and 70 °C, effectively addressing cross-sensitivity issues due to its low-temperature dependence. The miniature two-path structure of the MTP-MZI opened new opportunities for developing advanced multidimensional and multiparameter measurement techniques in MZI-based fiber sensing applications.

In the study “Design of Magnetic Fluid-Enhanced Optical Fiber Polarization Filter”, Chen et al. introduced a novel technique for enhancing surface plasmon resonance (SPR) in photonic crystal fibers (PCFs) by filling their air holes with magnetic fluid (MF) and coating them with a layer of the gold film [19]. Simulation results revealed significant improvements in polarization performance: at wavelengths ranging from 1260 to 1675 nm, the minimum loss coefficient for the y-polarization mode increased by a factor of 4.7 compared to the unfilled PCF, while the x-polarization mode experienced a 0.45-fold increase. Building on this enhancement, a polarizing filter was designed with a core diameter of 9 µm. Numerical simulations demonstrated that this filter retains the core diameter of a standard single-mode fiber while achieving a broader bandwidth and a higher extinction ratio (ER). Furthermore, the extinction ratio can be fine-tuned at specific wavelengths by adjusting the external magnetic field, offering precise control and flexibility. This method showed promising potential for advanced SPR applications and polarization-dependent optical devices.

## 4. Displays

Three-color electrophoretic displays (EPDs) are gaining attention due to their low energy consumption and excellent reflective qualities [20,21]. However, during black-and-white image transitions, the unique driving characteristics of red particles often prevent them from reaching their target positions, leading to the formation of red ghost images. In the study “Optimized Driving Scheme for Three-Color Electrophoretic Displays Based on the Elimination of Red Ghost Images”, Jiang et al. used the finite element method (FEM) to create a three-dimensional model, analyzing the motion behavior of electrophoretic particles to address this challenge. A novel driving scheme was proposed to precisely position red particles and eliminate red ghosting [22]. This approach involved optimizing the pixel-erasing process and incorporating a high-frequency oscillating voltage. The experimental results demonstrated that the new scheme reduced red ghost by 8.57% while enhancing white image brightness by 17.50%. These improvements significantly enhanced the performance of three-color EPDs, paving the way for their broader use in high-reflectivity and high-quality display applications.

The electrowetting display (EWD) is an advanced electrowetting-on-dielectric (EWOD) device capable of producing a paper-like display effect under electric field control. In this microfluidic system, grayscale stability can be influenced by factors such as material properties, device structure, and electric field distribution. In the paper “Optimizing Bipolar Reset Waveform to Improve Grayscale Stability in Active Matrix Electrowetting Displays”, Zhang et al. investigated the impact of driving voltage polarity on oil backflow to enhance the grayscale stability of active matrix electrowetting displays (AM-EWDs). An optimized inter-frame bipolar reset driving waveform was developed based on the driving principles of AM-EWDs to mitigate oil backflow [23]. This waveform stabilized the oil state by periodically releasing trapped charges in the dielectric layer through a reverse voltage. Additionally, the feed-through voltage effects on pixel driving voltage were addressed by compensating the driving voltage on a common electrode. A performance evaluation on a 6-inch AM-EWD driving platform showed that, compared to a conventional unipolar reset waveform, the oil backflow speed was reduced by 2.70 a.u./s and the standard deviation of luminance decreased by 11.24 a.u. These results demonstrate that the proposed driving waveform effectively suppresses oil backflow and minimizes luminance fluctuations, improving overall display performance.

## 5. Lenses

In the paper “Design and manufacture of 30-degree projection lens for augmented reality waveguide”, Sun et al. developed a projection lens with a 30-degree field of view for augmented reality (AR) glasses, incorporating a waveguide combiner optimized for a 0.35-inch LCoS panel [24]. The lens featured an entrance pupil diameter of 14 mm, an effective focal length of 16.443 mm, and an F-number of 1.175. This study addressed four key aspects: the design of the optical projection lens, a tolerance analysis for lens manufacturing and assembly, resolution testing of the projection lens, and an evaluation of AR system resolution for the panel images projected through the lens. Post-manufacturing tests revealed a central field image quality of 57 cycles/mm with an angular resolution of 33 pixels per degree (PPD). For the 0.7 fields, the image quality was measured at 40.3 cycles/mm, corresponding to an angular resolution of 23 PPD. Imaging performance tests using a diffraction-based waveguide confirmed a resolution of 57 cycles/mm in the center area, maintaining an angular resolution of 33 PPD. These results validated the lens’ suitability for high-resolution AR applications.

## 6. Frequency Conversion

The frequency conversion process using periodically poled thin-film lithium niobate (PPTFLN) has become a vital element in quantum information and photonic signal processing [25]. Nanophotonic waveguides, with their strong optical mode confinement, significantly enhance the normalized conversion efficiency (NCE) compared to bulk lithium niobate [26]. However, the power conversion efficiency of these devices is constrained by nanoscale inhomogeneities in thin-film lithium niobate (TFLN), which introduce phase errors. In the paper, “Efficient second-harmonic generation in adapted-width waveguides based on periodically poled thin-film lithium niobate”, He et al. proposed a theoretical solution to address these phase errors through dispersion engineering [27]. By locally adjusting the waveguide width at regions with varying thicknesses, the design eliminated phase mismatches. The approach was applied to etched and loaded waveguides on PPTFLN, achieving ultrahigh second harmonic generation (SHG) power conversion efficiencies of 2.1 × 10^4^%W^−1^ and 6936%W^−1^, respectively, surpassing the results of similar works. The proposed method required only standard periodic poling with a single period, simplifying electrode fabrication and reducing the complexity of the poling process. This design also supported multiple waveguides without the need for individual poling configurations. Combining simplicity, scalability, and compatibility with existing micro- and nanofabrication technologies, this work provided a new pathway for highly efficient second-order nonlinear optical processes on PPTFLN platforms.

## 7. Energy Harvesting

In recent developments, two-dimensional (2D) transition metal dichalcogenides (TMDs), such as molybdenum disulfide (MoS_2_) and molybdenum diselenide (MoSe_2_), have emerged as effective materials for extracting holes from perovskite layers [28,29]. The work function of MoS_2_ can be tuned across a broad range, from 3.5 to 4.8 eV, by modifying factors such as the number of layers, their chemical composition, elemental doping, surface functionalization, and surface states, depending on the synthesis method. In the paper “Curcumin-assisted synthesis of MoS_2_ nanoparticles as an electron transport material in perovskite solar cells”, Murugesan et al. presented a novel approach to synthesizing MoS_2_ nanoparticles (NPs) from bulk MoS_2_ using a two-step process: (1) exfoliating bulk MoS_2_ into few-layer MoS_2_ with curcumin–cholesteryl-derived organogels (BCC-ED) and curcumin solution in ethylene diamine (C-ED) under sonication, and (2) subjecting the few-layer MoS_2_ to ultrasonication at temperatures between 60 and 80 °C, followed by washing with solvents [30]. The initial exfoliation process resulted in a few-layer MoS_2_, as confirmed by SEM analysis. Extended sonication and washing yielded uniform MoS_2_ NPs with a size of approximately 10 nm. Since both BCC-ED and C-ED produced similar results, C-ED was chosen for further production due to the ease of removing curcumin from the MoS_2_ NPs.

When these MoS_2_ NPs were used as an electron transport layer (ETL) in a device structure of FTO/ETL/perovskite absorber/spiro-OMeTAD/Ag, their efficiency was significantly improved. The MoS_2_ NPs-based ETL exhibited a power conversion efficiency (PCE) of 11.46%, with a short-circuit current density of 18.65 mA/cm^2^, an open-circuit voltage of 1.05 V, and a fill factor of 58.66%, measured at a relative humidity of 70 ± 10% (open-air conditions). These results suggest that the combined effect of curcumin and ethylene diamine (ED) played a crucial role in producing high-quality MoS_2_ NPs, which enhanced charge transport, reduced crystal defects and trap sites, and minimized charge recombination, leading to significant efficiency improvements.

Perovskite solar cells (PSCs), which combine organic and inorganic materials, are emerging as a promising alternative to traditional silicon-based solar cells. The development of efficient electron transport materials (ETMs) is critical to advancing PSC technology. Due to their excellent electronic properties and cost-effective production, PSCs have gained significant attention as a potential future solution for thin-film solar energy. However, PSCs with compact layers (CLs) often face challenges related to their long-term reliability and efficiency. The quality of the substrate beneath the perovskite layer plays a crucial role in its growth rate and overall performance. As a result, there has been growing interest in modifying substrates with electron transport layers to improve the stability and efficiency of PSCs. In the review paper “The role of optimal electron transfer layers for highly efficient perovskite solar cells—A systematic review”, Vanaraj et al. reviewed the modifications made to electron transport layers (ETLs) in PSCs and their influence on the development of reliable and high-performance devices [31]. The paper also discussed the role of ETLs in achieving larger particle sizes, enhanced crystallization, and a more uniform morphology in perovskite films—factors that contributed to the long-term stability and efficiency of PSCs. Additionally, challenges and potential research directions for advancing compact ETLs were addressed, highlighting the importance of improving sustainability and maximizing clean energy output in future PSC developments.

## 8. Imaging

Axial resolution is a critical feature of a microscope, as it enables the efficient distinction of details along the longitudinal axis. A high axial resolution is generally preferred for precise imaging. However, when examining thick samples that do not involve laterally overlapping information, a lower axial resolution can be advantageous, as it allows for the simultaneous recording of data from multiple planes in a single camera shot, eliminating the need for mechanical refocusing between planes. In the paper “Extending the depth of focus of an infrared microscope using a binary axicon fabricated on Barium Fluoride”, Han et al. non-invasively enhanced the focal depth of an infrared microscope by incorporating a binary axicon made of barium fluoride, positioned close to the sample [32]. The initial imaging results of thick and sparse silk fibers demonstrated an extended focal depth, accompanied by a minor reduction in lateral resolution and an increase in background noise.

## 9. Optical Beam Emitters

Light beams with orbital angular momentum (OAM) are employed in various scientific and engineering fields, including microscopy, laser material processing, and optical tweezers. Accurate control of the topological charge is essential for effectively utilizing vortex beams in areas such as optical communication, information encoding, and sensor systems. In the paper “Micro-ring resonator-based tunable vortex beam emitter”, Bakirova et al. introduced a novel technique for optimizing an emitting micro-ring resonator (MRR) to generate vortex beams with adjustable OAM orders [33]. The MRR was composed of a ring waveguide with periodic structures coupled to a bus waveguide. The resonator’s tunability was enabled by the phase change material Sb_2_Se_3_, which was deposited onto the ring. This material underwent a transition from amorphous to crystalline phases, altering its refractive index. In the amorphous phase, the refractive index was 3.285, while in the crystalline phase, it increased to 4.050 at an emission wavelength of 1550 nm. This refractive index change was utilized to control the topological charge of the vortex beam. The spacing between the bus waveguide and the ring waveguide, the bending angle, and the width of the bus waveguide were optimized. The optimization criterion focused on maximizing the energy flux density radiated by the resonator. Numerical simulations confirmed the effectiveness of this method. This approach can be applied to optimize optical beam emitters carrying OAM for a range of applications.
